# Transcriptome analysis reveals key genes involved in the resistance to *Cryphonectria parasitica* during early disease development in Chinese chestnut

**DOI:** 10.1186/s12870-023-04072-7

**Published:** 2023-02-06

**Authors:** Xinghua Nie, Shuqing Zhao, Yaqiong Hao, Si Gu, Yu Zhang, Baoxiu Qi, Yu Xing, Ling Qin

**Affiliations:** 1 College of Forestry, Beijing Forestry University, Beijing, China; 2 College of Plant Science and Technology, Beijing University of Agriculture, Beijing, China; 3 Pharmacy and Biomolecular Science, Liverpool John Moores University, Liverpool, UK

**Keywords:** Chestnut blight, Chinese chestnut, Transcriptome analysis, Differentially expressed genes (DEGs), JA biosynthesis and metabolic pathway

## Abstract

**Background:**

Chestnut blight, one of the most serious branch diseases in *Castanea* caused by *Cryphonectria parasitica*, which has ravaged across American chestnut and most of European chestnut since the early twentieth century. Interestingly, the Chinese chestnut is strongly resistant to chestnut blight, shedding light on restoring the ecological status of *Castanea* plants severely affected by chestnut blight. To better explore the early defense of Chinese chestnut elicited in response to *C. parasitica*, the early stage of infection process of *C. parasitica* was observed and RNA sequencing-based transcriptomic profiling of responses of the chestnut blight-resistant wild resource ‘HBY-1’ at 0, 3 and 9 h after *C. parasitica* inoculation was performed.

**Results:**

First, we found that 9 h was a critical period for Chinese chestnut infected by *C. parasitica*, which was the basis of further study on transcriptional activation of Chinese chestnut in response to chestnut blight in the early stage. In the transcriptome analysis, a total of 283 differentially expressed genes were identified between T9 h and Mock9 h, and these DEGs were mainly divided into two clusters, one of which was metabolism-related pathways including biosynthesis of secondary metabolites, phenylpropanoid biosynthesis, amino sugar and nucleotide sugar metabolism, and photosynthesis; the other was related to plant-pathogen interaction and MAPK signal transduction. Meanwhile, the two clusters of pathways could be connected through junction among phosphatidylinositol signaling system, phytohormone signaling pathway and α-Linolenic acid metabolism pathway. It is worth noting that genes associated with JA biosynthesis and metabolic pathway were significantly up-regulated, revealing that the entire JA metabolic pathway was activated in Chinese chestnut at the early stage of chestnut blight infection.

**Conclusion:**

We identified the important infection nodes of *C. parasitica* and observed the morphological changes of Chinese chestnut wounds at the early stage of infection. In response to chestnut blight, the plant hormone and MAPK signal transduction pathways, plant-pathogen interaction pathways and metabolism-related pathways were activated at the early stage. JA biosynthesis and metabolic pathway may be particularly involved in the Chinese chestnut resistance to chestnut blight. These results contributes to verifying the key genes involved in the resistance of Chinese chestnut to *C. parasitica*.

**Supplementary Information:**

The online version contains supplementary material available at 10.1186/s12870-023-04072-7.

## Background

*Castanea* is an economically important tree in the world [1]. At present, seven species of *Castanea* plants are recognized, and there are significant differences in the resistance to chestnut blight among *Castanea* species [2]. Since the twentieth century, the once-dominant European and American chestnuts have been severely devastated by *Cryphonectria parasitica* (Murr.) Barr., causing an overwhelming crisis in European chestnut production and an endangered state of American chestnut throughout North America. However, *Castanea* plants of East Asia suffered less damage, especially Chinese chestnut and Japanese chestnut show significant resistance to chestnut blight [2, 3]. Therefore, East Asia chestnuts are kind of pivotal source of resistance to chestnut blight.

Chestnut blight is one of the world-famous forest diseases and is a typical example of plant disease that destroyed a dominant tree species [4]. The pathogen was first discovered in the New York Zoo in 1904 [5]. Over less than half a century, it almost destroyed the American chestnut trees in the entire territory. It was estimated approximately 4 billion trees involved in these catastrophes [6]. At present, the species in the wild is mainly new seedlings grown from the base of the dead chestnut trees. Genetic analysis of *C. parasitica* also revealed that due to the widespread introduction of Japanese chestnut in North America, *C. parasitica* had widely spread into North America [7]. In European, the *C. parasitica* introduced from North America was discovered in northern Italy in 1938, resulting in their widespread death [7, 8]. Luckily, the emergence of different strains of dsRNA hypovirus played a positive role in the widespread reduction in *C. parasitica* virulence and has allowed the European chestnuts to remain commercially viable in European [9]. Asian species (*C. crenata*, *C. henryi*, *C. mollissima* and *C. seguinii*) are generally resistant to *C. parasitica*, with strong immune activity. The reason is possibly co-evolution with *C. parasitica* in history [9–11]. Resistant species infected by *C. parasitica* commonly develop superficial lesions or ulcers on parts of the trunk and survive with burl at the infestation [12, 13]. In contrast, European (*C. sativa*) and American (*C. dentata* and *C. pumila*) species are highly susceptible to this pathogen. Once infected, they are killed [10, 12]. But to date, there has been no chestnut resource completely immune to *C. parasitica*, and the incidence rate even in China is as high as 60% in recent years, especially in chestnut orchards in southern China due to improper management and continuous environmental changes [14]. The infected chestnut trees grew weakly, which seriously affects chestnut production [14].

To restrict the further expansion of chestnut blight, researchers had been seeking various ways in the past few decades. A naturally occurring double-stranded RNA (dsRNA) hypovirus that can reduce *C. parasitica* virulence was discovered and widely applied in production across Europe [15, 16]. Since then, the chestnut blight of European chestnuts has been controlled. Unfortunately, it failed in North America because of various vegetative incompatibility types of chestnut blight [17, 18]. Simultaneously, some large-scale experiments were carried out to reduce the impact of chestnut blight worldwide, including genetic engineering, hybridization, and the excavation of disease-resistant genes [19–22]. Among them, researchers tried to cross the American chestnut with resistant Asian chestnut plants. Unfortunately, the goal of combining disease resistance in Asian chestnuts with the growth and morphology of American chestnuts has not been achieved [23]. Some researchers turned to disease-resistant genes (loci). Kubisiak et al. identified three major blight resistance QTLs via constructing a genetic map [21]. The sequenced candidate genes in these QTLs were analyzed by Staton et al. in 2015, 15 of which were annotated with the “defense response”, including *TGA1*, *RGA3*, *PBS1*, *RD19a*, *DAZ-associated protein 1-like*, *Proteasome subunit alpha type-7*, etc. facilitating further study on blight resistance [22]. However, the population that has mapped the disease resistance is few, hence loci with minor effects may not be detected and the effect size of loci with significant effects may be exaggerated [24]. The SUNY College of Environmental Science and Forestry (SUNY-ESF) also made progress on generating loads of transgenic *C. dentata* plants with overexpression of OxO enhancing resistance to chestnut blight [25, 26]. Chinese chestnut is a resistant species with commercial and ecological values, however, the previous studies only focus on the evaluation of Chinese chestnut disease resistance and the screening of excellent disease resistance resources. Little is known about the temporospatial interaction between the Chinese chestnut and *C. parasitica*, not to mention how the Chinese chestnut mediates immunity in response to this stress. In this study, the critical period of infection (CPI) of Chinese chestnut responding to *C. parasitica* was confirmed based on observation of the infection process. The transcripts of the inoculated and non-inoculated pathogen were compared to identify DEGs. It will provide insight into the resistance of *Castanea* plants to chestnut blight by exploring the disease resistance characteristics of the Chinese chestnut.

## Results

### The infection process of *Cryphonectria parasitica* on Chinese chestnut branches

**Fig. 1:**
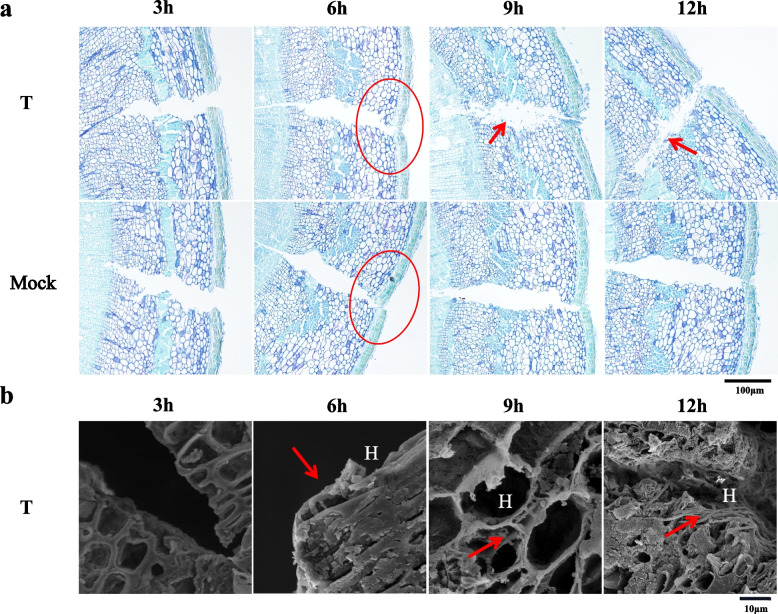
Morphological analysis of inoculated and non-inoculated Chinese chestnut branches at different times. **a** Microstructure of Chinese chestnut wounds at a different time (pathological tissue section method). Note: The red circle represents the indentation of the wound; The red arrow represents cell degradation at the wound. **b** Microstructure of Chinese chestnut wounds at a different time (scanning electron microscope observation method). Note: H represents the hyphae of *C. parasitica*

To clarify the critical infection period of Chinese Chestnut by *Cryphonectria parasitica*, the virulent strain ‘EP155’ was used to explore the infection process on a resistant wild Chinese chestnut resource ‘HBY-1’. The infection locations of *C. parasitica* and morphological contrast of the bark tissue structure for inoculated and non-inoculated branches were observed with electron microscopy (EM) at 3, 6, 9 and 12 hours, respectively. (Fig.1a, b and Additional file 1: Fig. S1). Three hours after inoculation, we found that the epidermis color deepened in both inoculated and non-inoculated branches, which was mainly the result of oxidation in the wounds. However, different from non-inoculated branches, a small number of hyphae could be observed to attach to the inoculated wound, which provided preparation for further infection. Six hours after inoculation, the epidermis of non-inoculated and inoculated branches began to lose water and slightly dented in the wounds, and hyphae could already be observed in the wounds of inoculated branches. Nine hours after inoculation, the hyphae increased in the epidermal cells of the inoculated branches, and the infection area was further expanded to the phloem and cambium. Simultaneously, we discovered that the cells in the phloem and cambium began to be degraded. Twelve hours after inoculation, it can be observed that a large number of white hyphae appeared in the wounds of the inoculated branches. The hyphae rapidly proliferate and accumulate in the wounds to form mycelium and the peripheral cells of the wounds were further collapsed.

Subsequently, the fungus growth of *C. parasitica* and the microscopic changes of epidermal cells after inoculation for 6, 9 and 12 hours were observed by scanning electron microscopy (SEM). Six hours after inoculation, the fungus could be observed in the epidermis and the wounds (Fig.1b), indicating that the fungus had begun to colonize, which was consistent with the semi-thin section and external observation. Surprisingly, 9 h after inoculation, the number of hyphae increased significantly, and a large number of hyphae existed around the cortical cells, phloem cells and cambium cells of the branches. Meanwhile, it was found that hyphae mainly transmitted in the cell junction or the space among cells, and more starch granules were produced in the cells with the infection of the fungus. At this time, the plant cells began to degrade, but the cell structure was still relatively intact. Twelve hours after inoculation, the epidermal cells in contact with the pathogen were further degraded and hyphae accumulated in large quantities.

Therefore, during the period of 0-6 h, the *C. parasitica* mainly proliferated at the wound site, and the epidermal cells were relatively stable. 9 h later, the fungus began to expand in the intercellular space, and the epidermal cells of the plant began to have obvious morphological and structural changes under the biological stress of *C. parasitica*, resulting in hypersensitivity reaction (Table 1). Hence, we determined that 9 h was the critical period for chestnut infection by *C. parasitica* (the period is abbreviated as CPI in this paper), which provided an important time node for us to further study the molecular mechanism of Chinese chestnut response from the beginning of infection to the time of structural changes in plant epidermal cells.

**Table 1:** Branch phenotypes at different time points after inoculation

Sample name	Characteristics	3 h	6 h	9 h	12 h
HBY-1	Phenotypic characteristics	Browning of the tissue at the bark wound	The wound begins to lose water and sag; a small number of hyphae can be observed attached to the bark	The browning area of the wound increases, and obvious white hyphae can be observed; obvious hyphae can be observed in the intercellular space of the phloem and cambium; some peripheral cells are degraded	The mycelium at the wound continued to proliferate, and the density of the mycelium increased; the degradation range expanded
	Site of infection	Bark epidermis	Bark epidermis	Bark epidermis, phloem and cambium	Bark epidermis, phloem and cambium

### RNA sequencing and mapping to the reference genome

To further understand the molecular mechanism of Chinese chestnut response at early chestnut blight infection, RNA-Seq was conducted using Illumina technology. According to the process of infection for Chinese chestnut branches by *C. parasitica*, the result revealed that the morphology of epidermal cells at the infection site kept a whole shape at 3–6 hours after infection, which was a stable period for the proliferation of *C. parasitica*. 9 h of infection was a key time point that the *C. parasitica* began to invade the intercellular space. At this time, it was also the time point that Chinese chestnut had an obvious stress response to the *C. parasitica*, and the cells at the infected wound of Chinese chestnut were degraded. Therefore, 0, 3 and 9 hours were selected as the three key periods for analyzing the response mechanism of chestnut to the parasitism of *C. parasitica*. In this experiment, total RNA was extracted from Chinese chestnut branches at 0 hour (without a scratch), 3 and 9 hours after non-inoculation (scratch), 3 and 9 hours after inoculation (scratch and inoculation), and 15 cDNA libraries were constructed (three repetitions per tissue, including 0 hours without *C. parasitica*: Mock0h-1, Mock0h-2, and Mock0h-3; 3 hours without *C. parasitica*: Mock3 h-1, Mock3 h-2, and Mock3 h-3; 9 hours without *C. parasitica*: Mock9 h-1, Mock9 h-2, and Mock9 h − 3; 3 hours with *C. parasitica*: T3 h-1, T3 h-2, and T3 h-3; 9 hours with *C. parasitica*: T9 h-1, T9 h-2, and T9 h-3). After the raw data were removed and trimmed, we obtained 17–32 million clean reads for each library. The Q20 and Q30 were > 97.61% and > 93.12%, respectively. In addition, the GC contents were 43.57–44.84% among all samples (Additional file 2: Table. S1), suggesting that the sequencing was highly accurate. The public Chinese chestnut genome (PRJNA527178) was used as the reference genome. The majority of the reads from Mock0h (91.81–92.43%), Mock3 h (91.96–93.09%), Mock9 h (92.33–92.73%), T3 h (89.90–92.66%), and T9 h (86.84–87.57%) were successfully aligned to the reference genome.

### Identification and trend characteristics analysis of DEGs

**Fig. 2:**
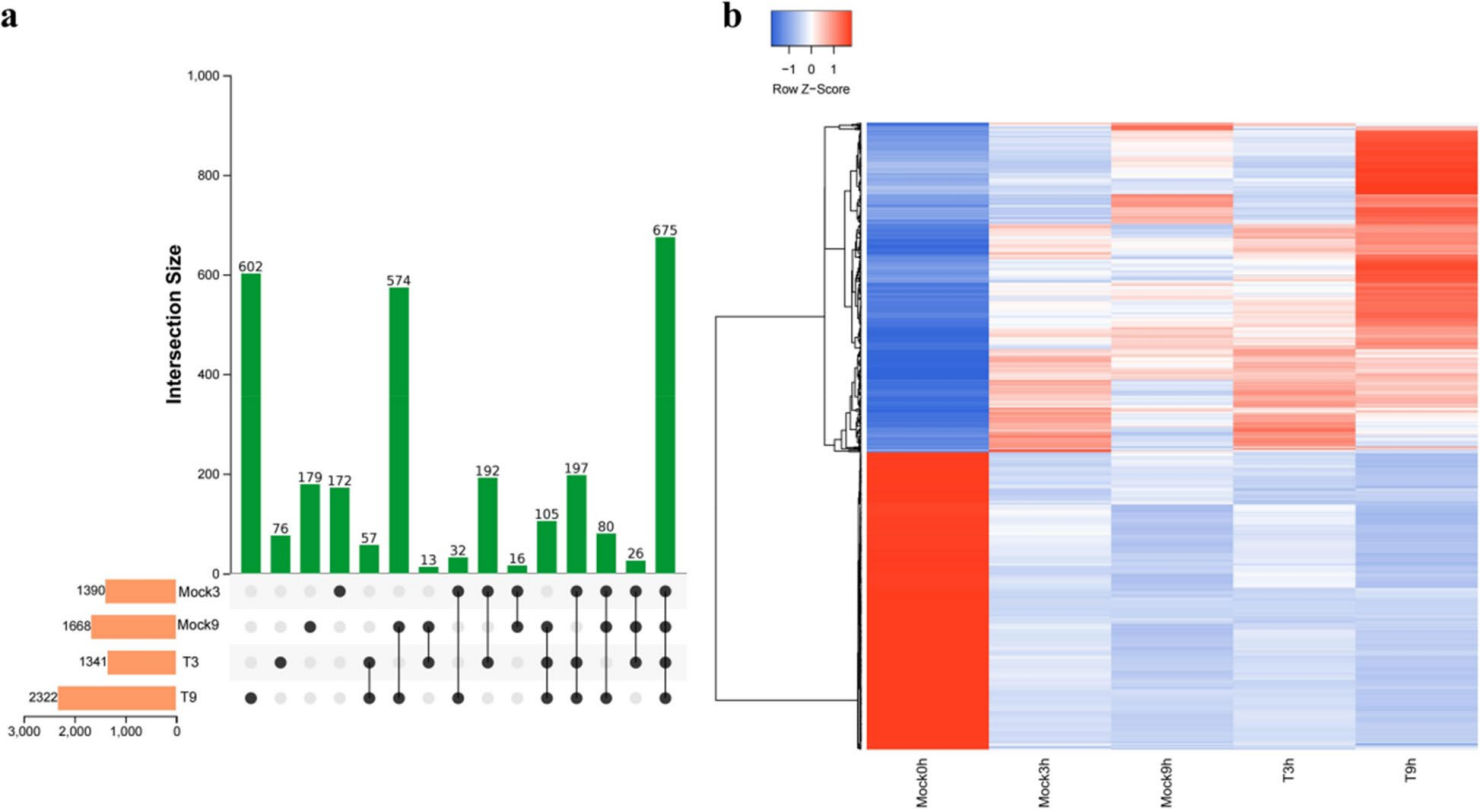
DEGs in inoculated and non-inoculated barks. **a** Venn diagram of DEGs among groups, **b** thermographic analysis of DEGs at the transcriptome level at 9 hours after inoculation

Chestnut blight is usually acquired through contact with wounds, followed by the appearance of large ulcers on the branch. Therefore, the chestnut plant has to face the damage from the germs and also the damage caused by the ulcers. To analyze which genes are activated in the infection process, we selected samples that were punched with non-inoculated branches as a Mock group. Due to the influence of the above two aspects, DEGs (differentially expressed genes) were extracted according to their expression levels in our sequenced samples and identified at Mock3 h and Mock9 h, and we found 1390 genes (762 up-regulated genes/628 down-regulated genes) and 1668 genes (626 up-regulated genes/1042 down-regulated genes), respectively (Fig. 2a). In contrast, the DEGs at T3 h and T9 h were 1341 genes (787 up-regulated genes/554 down-regulated genes) and 2322 genes (1051 up-regulated genes /1271 down-regulated genes), respectively (Fig. 2a). There was a gradual increase in the genes that responded across and within groups over time. For example, the total number of DEGs in T9 h was 654 genes more than that in Mock9 h, and there were 574 DEGs shared between the two groups, accounting for 34.41% (Mock9 h vs Mock0h) and 24.72% (T9 h vs Mock0h) of their respective DEGs (Fig. 2a). Within-group, Mock9 h was 308 more than Mock3 h responsive DEGs, while T9 h was nearly twice as many as T3 h responsive DEGs, and the inoculation group showed more significant changes. To understand the specific genes that responded to *C. parasitica*, we screened and enriched the DEGs of T3 h vs Mock3 h and T9 h vs Mock9 h, among which there were only 63 DEGs in T3 h vs Mock3 h (27 up-regulated genes, 36 down-regulated genes), and the DEGs were distributed in pathways including biosynthesis of secondary metabolites, MAPK signaling pathway, phosphatidylinositol signaling system, phenylpropane biosynthesis and plant-pathogen interaction and so on (Additional file 3: Fig. S2). The differential genes of T9 h vs Mock9 h had a total of 283 genes (91 down-regulated genes and 192 up-regulated genes). The results of KEGG enrichment revealed that the DEGs are mainly enriched in photosynthesis, phosphatidylinositol signaling system, α-linolenic acid metabolism, MAPK signaling pathway, plant-pathogen interaction, metabolic pathways, amino sugar and nucleotide sugar metabolism, isoquinoline alkaloid biosynthesis and isoflavone biosynthesis pathways. In addition, the identified DEGs were analyzed by cluster analysis and thermographic analysis at different time points (Fig. 2b).

**Fig. 3:**
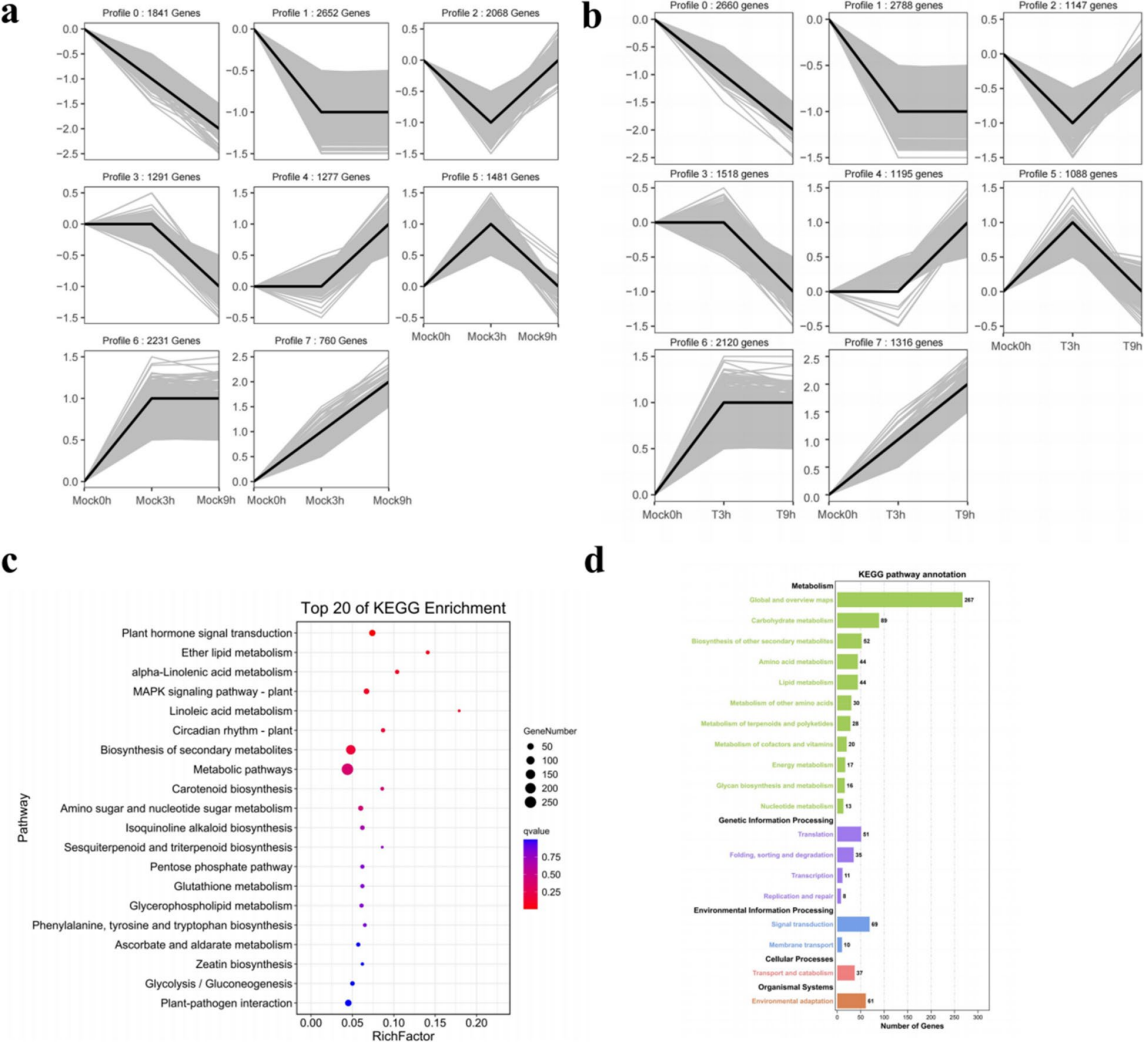
Analysis of trend genes for different trend characteristics of Mock0h-T3 h-T9 h and Mock0h-Mock3 h-Mock9 h. **a** The number of trend genes for different trend characteristics of Mock0h-T3 h-T9 h. **b** The number of trend genes for different trend characteristics of Mock0h-Mock3 h-Mock9 h. **c** KEGG pathway enrichment trend of common DEGs between Mock0h-T3 h-T9 h and Mock0h-Mock3 h-Mock9 h. **d** Functional annotations of common DEGs between Mock0h-T3 h-T9 h and Mock0h-Mock3 h-Mock9 h

Then, the DEGs of inoculated and non-inoculated samples were categorized according to the trend characteristics of gene expression at three-time points (Fig. 3a, b). Trend analysis was performed on branches at Mock0h-T3 h-T9 h, and a total of 13,832 DEGs were identified. Among them, 1316 genes showed a continuous upward trend with the extension of infection time, and 2660 genes showed a continuous downward trend. Among the genes showing an upward trend, 1134 genes were annotated in KEGG. The enrichment analysis mainly focused on biosynthesis of secondary metabolites, phenylpropane metabolism, phenylpropanoid biosynthesis, starch and sucrose metabolism, amino acid synthesis, isoquinoline alkaloid biosynthesis, beta-Alanine metabolism and tropane, piperidine and pyridine alkaloid biosynthesis related metabolic pathways, ribosome synthesis-related translation pathways and the environmental adaptation pathway of plant-pathogen interaction, plant hormone signal transduction and MAPK signaling pathway. The downward trend genes were mainly enriched in photosynthesis, plant-pathogen interaction, plant hormone signal transduction, α-linolenic acid metabolism, MAPK signal pathway and endocytosis in KEGG (Additional file 4: Fig. S3). We were concerned that both the downward and upward trends contain plant-pathogen interaction, plant hormone signal transduction and MAPK signaling pathway, which are significantly related to plant response to diseases. The genes of upward trend mainly included FLS2, BAK1, PR1, FRK1, EFR, WRKY, PTI1, PTI5, CNGC, RPS2, while the genes of downward trend included HSP90; SAUR, GID, CALM, RBOH, EDS (Additional file 5: Table. S2). A total of 13,601 DEGs were identified in the trend analysis in Mock0h-Mock3 h-Mock9 h (Fig. 3a), of which 760 genes showed a continuous upward trend with the extension of infection time, and 1841 genes showed a downward trend. Among them, 1538 genes were overlapped with Mock0-T3 h-T9 h, and these DEGs were mainly enriched in plant hormone signal transduction, ether lipid metabolism, α-linolenic acid metabolism, linoleic acid metabolism, MAPK signaling pathway, biosynthesis of secondary metabolites, metabolic pathways, amino sugar and nucleotide sugar metabolism and circadian rhythm pathways (Fig. 3c, d). These shared genes play an important role in Chinese chestnut in response to *C. parasitica* and mechanical damage.

### Genes and pathways in response to *C. parasitica* infection

**Fig. 4:**
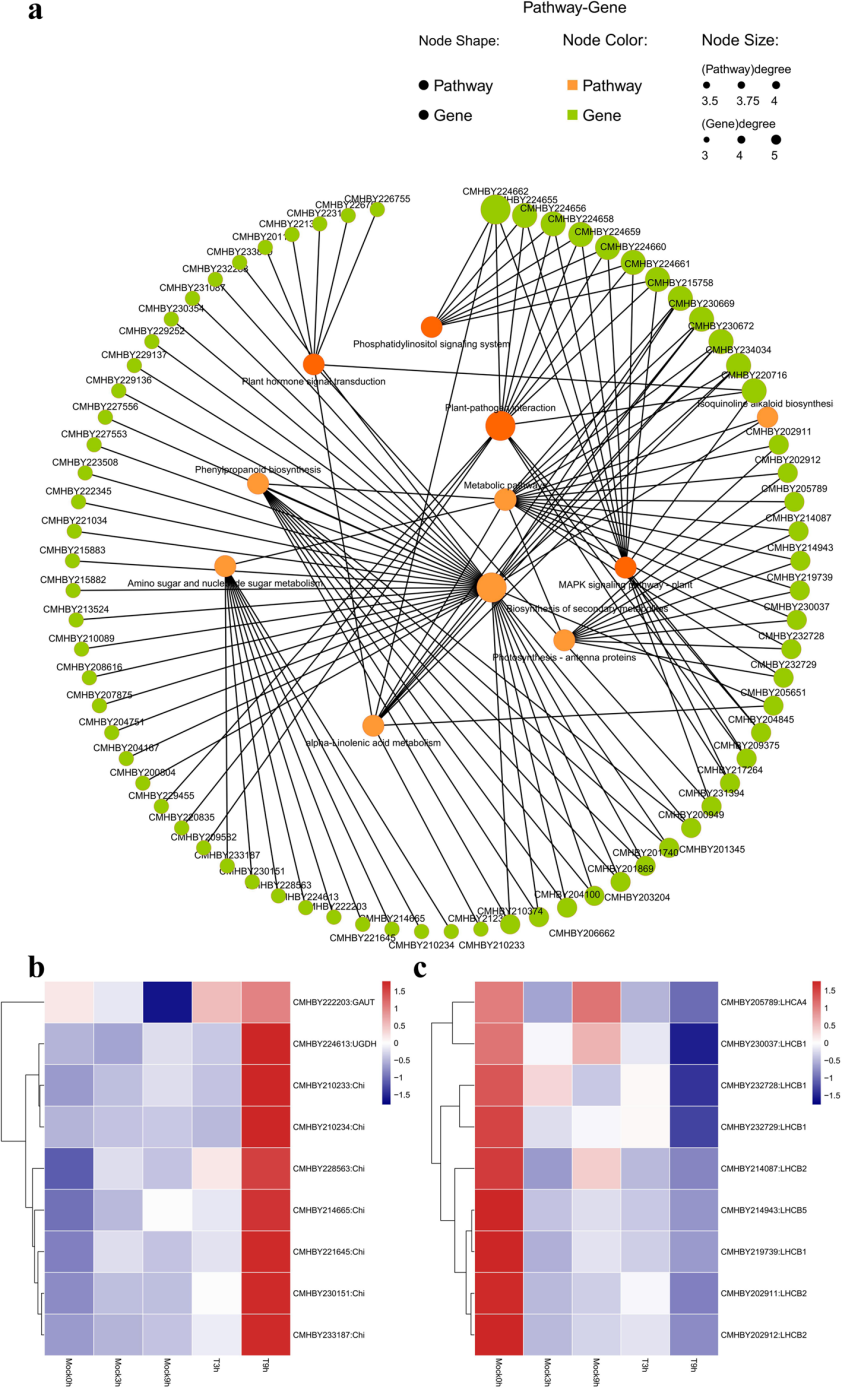
Pathway and gene network relationship of Chinese chestnut in response to chestnut blight fungus. **a** Pathway and gene network relationship response to chestnut blight fungus; **b** The heatmap of differential genes in the Photosynthesis-antenna protein pathway; **c** Heatmap of DEGs in Amino sugar and nucleotide sugar metabolism

In general, KEGG enrichment analysis results only reveal the enrichment degree of the individual pathway, which fail to provide better correlations among pathways, leading to the fragmentation of enrichment information. Here, the comparative transcriptional data of T3 h vs Mock3 h and T9 h vs Mock9 h were firstly used to screen 10 major pathways specifically responsive to chestnut blight infection. Whereafter, the data was comprehensively analyzed from the perspective of interactions among pathways (Fig. 4a). The results showed that 10 pathways were divided into two clusters. One cluster was metabolism-related pathways centered on biosynthesis of secondary metabolites, phenylpropanoid biosynthesis, amino sugar and nucleotide sugar metabolism, and photosynthesis, the other cluster was related to plant-pathogen interaction and MAPK signal transduction. Meanwhile, the two clusters of pathways could be connected through the relationship between phosphatidylinositol signaling system, phytohormone signaling pathway and α-Linolenic acid metabolism pathway.

When plants are stressed by pathogens, plants often respond through the innate immune system, metabolism, and phytohormone-mediated defense responses. Among them, the innate immunity of plants mainly includes PAMP-triggered immunity (PTI) and effector-triggered immunity (ETI). Therefore, in order to characterize the innate immune response of Chinese chestnut at the early stage, we focused on the genes of the pathways involved in plant-pathogen interaction and MAPK signaling pathway. Subsequently, we further annotated the DEGs of the relevant pathways at 3 h and 9 h after inoculation in the KEGG databases. At 3 h after inoculation, a total of 13 genes were differentially expressed in the MAPK signaling pathway, including 4 WRKY, 2 CALM, 2 FRK, 2 ERF, 2 PYL and 1 MYC. In the plant-pathogen interaction pathway, a total of 16 genes were differentially expressed, including 2 CALM, 4 CML, 3 CPK, 1 CNGC, and 2 FRK genes, revealing that the cationic channel was activated and Ca^2+^ was effused in large quantities and PTI reaction has begun to play an important role. At 9 h after inoculation, in addition to the continued functioning of MAPK signaling pathway, RBOHD was also activated in this process, foreboding ROS began to accumulate and chestnut bark cells appear hypersensitivity response (HR) at this stage, which is consistent with the appearance of apoptosis at the wound site in microscopic observations. Simultaneously, the PR and EDS disease process genes were also activated, and the sensitivity of Chinese chestnut to *C. parasitica* was enhanced, which in turn stimulated a stronger autoimmune response. Unfortunately, the resistance gene (R gene) related to the second layer of defense (ETI) was not found in the DEGs. We speculated that due to the relatively short infection time, the ETI response of Chinese chestnut may not be activated during the 9 h inoculation process, and the R gene remained silent.

After 9 h of inoculation, in the metabolism-related pathways, we found that the DEGs were mainly enriched in the following four pathways: photosynthesis-antenna protein energy metabolism, the amino sugar and nucleotide sugar metabolism, carbohydrate metabolism, and the α-linolenic acid metabolism and phenylpropanoid biosynthesis. Among them, a total of 9 down-regulated LHCA and LHCB genes were identified in the photosynthetic metabolic pathway (Fig.4b), suggesting that the photosynthesis of Chinese chestnut was weakened under the stress of *C. parasitica*. The genes of other enriched metabolic pathways, such as amino sugar and nucleotide sugar metabolism pathways, α-linolenic acid metabolism pathway and phenylpropanoid biosynthesis pathway, were all up-regulated. Among them, 9 genes were enriched in amino sugar and nucleotide sugar metabolism pathways (Fig.4c), including 1 UGDH, 1 GAUT and 7 chitinase genes. A total of 8 genes were enriched in the phenylpropanoid biosynthesis pathway, namely 1 cinnamyl-alcohol dehydrogenase gene, 1 feruloyl-CoA 6-hydroxylase gene, 4 peroxidase genes, 1 caffeoyl-CoA O-methyltransferase gene and 1 trans-cinnamate 4-monooxygenase gene.

In the plant hormone signal transduction pathway, we found that almost common genes of the phytohormone signal transduction pathway were identified at 3 h and 9 h after inoculation, mainly including 2 ERF, 3 SAUR, 1 ARR-B, 2 PYL, 1 GID, 1 MYC transcription factor and 1 BRI. In terms of disease resistance-related hormone biosynthesis pathways, there were 1 DEG (CMHBY209205), 2 DEGs (CMHBY206784 and CMHBY208616), and 9 DEGs (CMHBY205371, CMHBY206131, CMHBY202419, CMHBY210933, CMHBY204167, CMHBY212531, CMHBY201069, CMHBY214917, CMHBY218056) and 3 DEGs (CMHBY231087, CMHBY208478, CMHBY201439) in the biosynthesis pathways of gibberellins (GAs), abscisic acid (ABA), ethylene (ET)) and brassinosteroids (BR) were identified. Furthermore, it is worth noting that most of the key genes on the Jasmonic acid (JA) metabolic pathway exhibited differential expression patterns, including hydroperoxide dehydratase (AOS), acyl-CoA oxidase (ACX), alpha-dioxygenase (DOX), 12-oxophytodienoic acid reductase (OPR), jasmonate O-methyltransferase (JMT), phospholipase A (DAD), JAR, enoyl-CoA hydratase/3-hydroxyacyl-CoA dehydrogenase (MFP), etc. The results of expression thermogram showed that except for CMHBY231087 and CMHBY220949, the expression of the other genes decreased significantly (Additional file 6: Fig. S4). All of them increased with the prolongation of infection time, which indicated that the hormone-mediated defense response played an active role in the response of chestnut to *C. parasitica*.

### Real-time qPCR validation

**Fig. 5:**
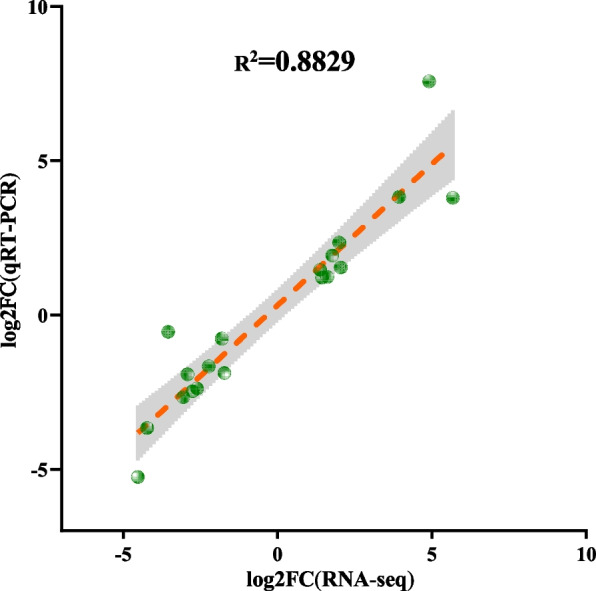
Correlation between result of qRT-PCR and RNA-Seq expression

To verify the reliability of the transcriptome data, real-time fluorescence quantitative technology was used to analyze randomly selected 20 genes that were differentially expressed at the transcriptional level (Additional file 7: Table. S3). *Actin* was used as an internal reference gene to determine and calculate the relative expression levels of the screened genes. The results confirmed that the transcriptional changes of these 20 genes had consistent characteristics with the fold changes we observed in the transcriptome analysis (Fig. 5).

## Discussion

Chinese chestnut is one of the most potential resources for improving the disease resistance of chestnut plants with weak disease resistance. In previous studies of chestnut blight progression and host response, although the information is presented in detail, it should be noted that these observations were carried out in only a few studies on a limited number of specimens [8, 27, 28]. Especially from various habitats, chestnut plants may have distinct responses to chestnut blight. To detect how this fungus infects the host specimens at the early stage. We first used in vitro experiment to analyze the process of infecting Chinese chestnut branch with *C. parasitica*. The results showed that *C. parasitica* had begun to prolificate on the wound bark epidermis and expanded rapidly between the bark and cambium cells at 3–6 hours.

At 9 h, a compact hyphal layer in the cambium was formed as the hyphae expanded through the chestnut cortex, phloem, and cambium cells, and massive accumulation. Subsequently, the hyphae continued to grow laterally in the intercellular space of the host, and the cell wall of the host bark also begins to be degraded. The characteristics of *C. parasitica* proliferation at 9 h was usually considered to be an important node for canker formation and enlargement of the lesion area [28, 29]. In addition, more starch granules were also observed near the hyphae at 9 h. It’s tempting to assume that these starch granules may act to hinder the further expansion of the pathogenic hyphae in the cell. Hence, we further explored this node underlying mechanism response to pathogen infecting Chinese chestnut.

Plants are generally attacked by a variety of microbial pathogens throughout the whole life cycle [30]. Despite the fact that they lack the adaptive immune system like mammals, plants can use their innate immunity to acquire resistance or induce systemic resistance throughout the plant production system in response to pathogens [31]. Plants sense, recognize and respond to pathogens through surveillance of self/non-self perception, damaged self, and altered self as danger signals, a mechanism by which plants achieve resistance to most pathogens (or potential pathogens) [32]. In the process of competing with various pathogenic bacteria, plants have gradually evolved an interacting two-layer innate immune system, the first layer consists of pattern recognition receptors (PRRs) on the cell membrane surface by recognizing pathogen-associated molecular patterns (PAMPs) derived from the surface of pathogenic bacteria or damage-associated molecular patterns (DAMPs) derived from plants, then an immune response was triggered in plants, termed PTI; In the second layer, the plant recognizes the corresponding pathogenic effector (Effector) through a specific NBS-LRR (Nucleotide-binding site-leucine rich repeat) class of R proteins and induces an immune response called ETI [33]. In the present study, the DEGs of inoculated and non-inoculated chestnut branches for 3 h and 9 h was performed to better identify Chinese chestnut responding to pathogens during the CPI period. It enfolds that the DEGs focus on these pathways including photosynthesis-antenna proteins, alpha-Linolenic acid metabolism, MAPK signal transduction pathway, plant-pathogen interaction, phenylpropanoid biosynthesis, phytohormone signal transduction, biosynthesis of secondary metabolites, metabolic pathways and other pathways related to plant stress. As disclosed in the transcriptome analysis, DEGs were mainly concentrated in the genes associated with the PTI response because of the time point we explored when the CPI response at the early stage is active instead of the ETI response. After Chinese chestnut recognizing *C. parasitica* at 3 h, CNGC, CALM/CML, WRKY22/WRKY33 transcription factor, and FRK genes were up-regulated, thus calcium channels were activated at this time, leading to intracellular Ca2+ accumulation. When time passed to 9 h regarded as the pivotal point, cells are hypersensitive due to ROS production and accumulation mediated further by the calcium-dependent protein kinase (CPK) and RBOHD genes besides the genes mentioned above which were consistent with the observation of the microstructure. The MAPK cascade also plays a central role in defense signaling against pathogens [34]. 9 h after inoculation, PR1, ERF1, MYC transcription factor and FRK involved in MAPK cascades were stimulated. It can be seen from the above results that the PTI response in Chinese chestnut is orchestrated in the presence of *C. parasitica* where the Ca^2+^ regulation signal and MAPK pathway are required, laying a foundation for its further defense response.

The co-evolutional characteristic of plants and pathogens is an intense competition between penetrating and strengthening cell walls. Pathogens degrade plant cell walls through synthesizing proteases such as cellulase to infect the host. Simultaneously, to resist damage from pathogens, plants strengthen cell walls via a reverse way like synthesizing enzyme inhibitors and accumulating callose and lignin [35]. Likewise, plants entail chitinases and β-1-3-glucanases to degrade pathogenic carbohydrates [36, 37] of which Chitinases are one of the most important inducible defense-related proteins by hydrolyzing chitin glycosidic bonds [38, 39]. In this study, we screened 7 genes encoding chitinases in the pathways of amino sugar and nucleotide sugar metabolism which were up-regulated, indicating that chitinases played an important role in early chestnut disease resistance. Similar results were shown by Barakat et al. (2012) that chitinases accumulation were more in ulcerated than in healthy tissue [40]. Vannini et al. (1999) demonstrated that chitinases were able to inhibit the *C. parasitica* strains growth to some extent [41]. Recent resistant transformed European chestnut lines were generated with overexpressing of the *CsCHI3* [42]. However, it remains to be seen whether these transformed lines can improve tolerance to chestnut blight in vitro. In conclusion, chitinases played an essential role at the early stage of the Chinese chestnut defending *C. parasitica*.

**Fig. 6:**
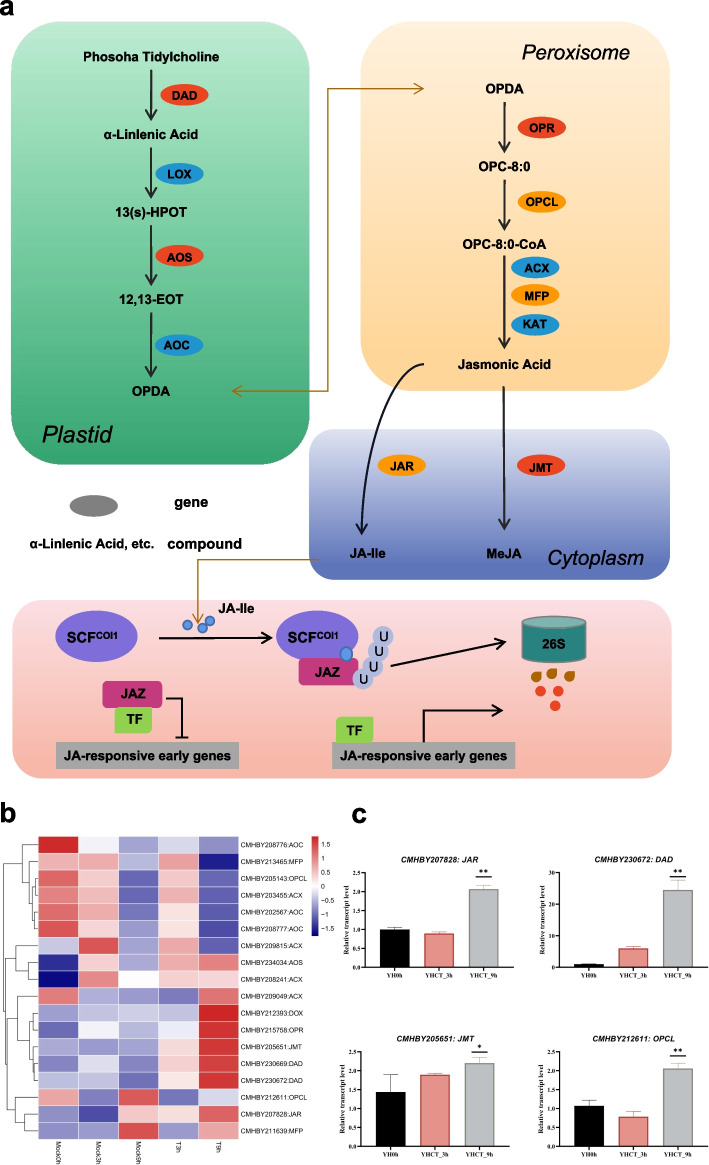
Schematic diagram of the Jasmonic acid biosynthesis and metabolic pathways. **a** Jasmonic acid biosynthesis pathway and related signal transduction in Chinese chestnut (this image was granted permission by KEGG), note: The ovals represent genes in the pathway diagram, the ovals marked in red are significantly up-regulated genes, the ovals marked in orange are up-regulated genes, and the ovals marked in blue down-regulated genes. **b** Heatmap Analysis of related genes in Jasmonic acid biosynthesis and metabolic pathways. **c** qRT-PCR relative expression levels of JMT, OPCL, DAD and JAR in disease-resistant cultivar ‘Yanhong’

Previous research has shown that salicylic acid (SA), jasmonic acid (JA), ethylene (ET), brassinosteroids (BR), gibberellins (GAs) and abscisic acid (ABA) were the main hormones involved in plant disease resistance. Of them, SA, JA and ET have been known as plant disease-resistant hormones so far [43–45]. After plant sensing, SA is depolymerized to form monomers and enter the nucleus to bind TGA transcription factors, thereby activating the expression of a series of WRKY transcription factors and PR genes, initiating downstream defense responses, and improving plant resistance to pathogens [46, 47]. Meanwhile, the SA signaling is antagonistic with auxin pathway in most cases [48]. We found PR expression in the SA signaling was significantly up-regulated since SA was accumulated in Chinese chestnut branches after pathogen inoculation, while the related genes in the auxin synthesis pathway were down-regulated, which was consistent with the above findings. ET is a gaseous phytohormone with a simple structure and induces hypersensitivity reactions (HR) in plant immune responses to pathogenic microorganisms and inhibits the expansion of pathogens [49]. In this study, the ERF gene showed an increasing regulation after pathogen infection. These agree with this observation that the cells at the wound appeared dead at 9 h. This hypersensitivity reaction may be caused by the accumulation of ET and ROS. Therefore, it was determined that Chinese chestnut were induced to appear HR in a short time, which can effectively resist further damage by *C. parasitica*. In addition, it is worth noting that more genes were significantly enriched in the linolenic acid metabolism pathway which is the key pathway of JA biosynthesis. JA and its derivatives are involved in a variety of plant stress responses, especially in plant defenses against insects and necrotrophic pathogens [44, 50]. Subsequently, we conducted a heat map analysis of the key genes in the biosynthesis and metabolic pathways and found that AOS, ACX, DOX, OPR, JMT, DAD, JAR, and MFP were all up-regulated, revealing that the entire JA metabolic pathway was activated in Chinese chestnut at CPI period (Fig.6a and b). The result was further verified in the known disease-resistant cultivar ‘Yanhong’ (Fig.6c). In previous research, JA and ET and SA signaling pathways were generally considered to function antagonistically in plant defense responses [51]. These phytohormone signalings pathways positively modulate Chinese chestnut resistance to chestnut blight in our transcriptomic analysis yet with a complicated but cooperated cross-relationship. Taken above, it is not only proved that SA, JA and ET can be required in the process of Chinese chestnut resisting *C. parasitica*, but also displays that the mechanism among these hormones is very complicated during this response. It remains to be investigated how these three signalings pathways interact with each other and if another hormone involves.

## Conclusion

In summary, we firstly determined 9 h representing the important infection nodes of *C. parasitica* via observation of the morphological wound changes infected at the early stage in Chinese chestnut. Then, the transcriptome datasets of inoculation and non-inoculation were designed and sequenced enclosing the key early disease resistance-related genes and pathways. This analysis displayed that JA, SA, ET and MAPK signaling pathways and plant-pathogen interaction pathways were mediated at 9 h after inoculation by *C. parasitica*. Importantly, the expression pattern of genes related to JA biosynthesis, phenylpropanoid biosynthesis, and chitinase biosynthesis resist the stress of *C. parasitica*. All these differentially expressed genes contribute to our more extensive and deeper understanding of the disease defense of Chinese chestnut and facilitate future breeding of chestnut blight resistant resources. These results provide important references for further understanding the defense of Chinese chestnut resistance to chestnut blight.

## Materials and methods

### Infection of chestnut blight on chestnut branches

Inoculations were done primarily with the strain of *C. parasitica* preserved in the College of Plant Science and Technology, Beijing University of Agriculture. The wild Chinese chestnut material in this experiment was grafted at the Chestnut Technology Experiment and Extension Station of Huairou District in 2015. Meanwhile, to reduce the errors caused by the environment and the growth state of branches, some criteria had been established, (1) the test branches can be healthy and non-damaged; (2) the test branches are biennial branch with a uniform diameter; (3) the branches of the mock group and treatment group were derived from the same branch group of a tree. Based on this, we preferentially selected a healthy biennial branch larger than 18 cm and sterilized spraying 75% ethanol. Then, the branch was aliquoted 2 groups of branches: one was Mock group, the other was Treatment(T) group. Subsequently, 5 mm wound were created by a scalpel where its depth reached to the cambium and its intervals were 3 cm. Meanwhile, creating the agar plugs with *C. parasitica* with a cork borer (diameter 6 mm) around the actively growing border of the fungal colony. After wounding, an inoculated agar plug was immediately placed directly on each wound as a treatment group and a non-inoculated agar plug (without *C. parasitica*) was also placed on each wound as a Mock group, and each group was repeated 3 times. The branches with agar plugs were then placed in a plastic tray lined with slightly damp paper towels. Trays were stored in an incubator with a temperature of 28 °C, a humidity of 70%, and illumination of 16 h/d.

### Sample preparation for semi-thin slices

Samples for microscopic observation were collected at 0 h, 3 h, 6 h, 9 h, and 12 h after inoculation, and then stored in fixative at 4 °C. The Chinese chestnut branch semi-thin slices were prepared as per Hao et al. (2022) [29].

### Sample preparation for scanning Electron microscopy

The inoculated parts of branches infected with *C. parasitica* were cut into small cylindrical pieces with a thickness of 0.5–1 mm. Cuts were placed into a 2 ml centrifuge tube filled with 1 mL fixative solution (70% tert-butanol: 40% formaldehyde: acetone: glycerin = 17:1:1:1, V/V/V/V). The samples were evacuated until there were no bubbles in the solution soaking the smples and the material sinked to the bottom of the centrifuge tube. They were stored in the refrigerator at 4 °C for 2 days. They were rerinsed with PBS buffer solution pH = 7.4 with 5–6 times, for 30 minutes each time. The cleaned chestnut branch samples were dehydrated by ethanol solutions with different concentration gradients (10, 30, 50, 70, 90, 95 and 100%) for 30 min, and was dehydrated by 100% ethanol twice. After dehydration, they were replaced with tert-butanol three times for 30 min each time at room temperature. Placed the replaced sample into a 2 ml centrifuge tube, then added an appropriate amount of tert-butanol, when tert-butanol just covered them,the samples were chilled at − 20 °C for 15 minutes. The samples were dried in freeze-dryer until tert-butyl alcohol was vanished. Samples were adhered to the sample table with conductive adhesive and then placed in a scanning electron microscope for observation and photography after powdered gold spraying.

### Total RNA extraction and Transcriptone sequencing library preparation

All bark tissues treated with EP155 for 3, 9 hours and tissues treated without EP155 for 0,3, 9 hours were sampled for RNA-Seq with three independent biological replicates for each. Total RNA was extracted using an E.Z.N.A.® Plant RNA Kit (OMEGA, Guangzhou, China) and the quantity and quality of RNA were evaluated by a NanoDrop spectrophotometer (NanoDrop Technologies, Wilmington, DE, USA). Subsequently, the RNA samples were subjected to strict quality control, mainly through the Agilent 2100 bioanalyzer: precise detection of RNA integrity. The mRNA was enriched by magnetic beads with Oligo (dT) for RNA-Seq. The constructed library was sequenced with Illumina Hi-Seq6000 (Illumina, San Diego, CA, USA).

### Transcriptome assembly and gene functional annotation

Firstly, Fast-QC was used for quality control analysis of RNA-SEQ sequences that were generated by Illumina sequencing platform (https://www.bioinformatics.babraham.ac.uk/projects/fastqc/). Using HISAT2 software to quickly and accurately align the quality control data with the reference genome of chestnut (https://www.ncbi.nlm.nih.gov/bioproject/PRJNA527178), and then using StringTie software to assemble new transcripts. After transcript assembly, we annotated and counted the new transcripts in five major databases including nonredundant (nr), Swiss-Prot, GO, Pfam and KEGG.

### Differential gene expression analysis

The analysis of DEGs was performed using the DESeq. The *P* values were adjusted using the Benjamini–Hochberg approach for controlling the FDR. Genes with an adjusted FDR < 0.01 identified by DESeq and log2 FPKM (fold change) ≥ 1.5 were considered to be differentially expressed. The Gene trend analysis, KEGG enrichment analyses of DEGs and pathway network analysis were performed using the OmicShare tools, a free online platform for data analysis (http://www.kegg.jp/kegg/path- way.html and https://www.omicshare.com/tools) [52–54].

### qRT-PCR analysis

To verify the accuracy of the screened genes, the total RNA of bark tissues was extracted at 3 h and 9 h after inoculation and at 0 h, 3 h and 9 h without inoculation, and the 20 differential expression genes were analyzed by qRT-PCR after reverse transcription (Additional file 7: Table. S3). The SYBR® premixed ExTaq™ II kit (TaKaRa, Japan) was used for real-time quantitative PCR (RT-qPCR). The data was normalized with the expression level of *Actin* as the internal control. Each condition of qRT-PCR has three biological replicates, and the relative expression levels of each gene were calculated. To further prove the role of JA metabolic pathway in chestnut resistance to chestnut blight, qRT-PCR also was performed for the genes related to this pathway (JMT, OPCL, DAD and JAR) using a known Chinese chestnut cultivar ‘Yanhong’ with great disease resistance. Specific primers designed according to the selected JMT, OPCL, DAD and JAR sequences are listed in the attached file (Additional file 8: Table. S4).

### Statistical analysis

The correlation analysis of the relative expression of qRT-PCR and the relative expression of transcription and the analysis of variance of the relative expression of qRT-PCR between different samples were calculated in GraphPad Prism 9.0.0 [55].

## Supplementary Information

**Additional file 1: Fig. S1.** Morphological analysis of inoculated (T) and non-inoculated (Mock) wounds of branches at 3 h, 6 h, 9 h and 12 h through stereomicroscopy.

**Additional file 2: Table. S1.** Data quality assessment of each sample.

**Additional file 3: Fig. S2.** Differentially expressed genes (DEGs) at different time points.

**Additional file 4: Fig. S3.** Differentially expressed genes (DEGs) at different trend characteristics at Mock0h-T3 h-T9 h. a: The genes showing an upward trend were annotated in KEGG; b: The genes showing an downward trend were annotated in KEGG.

**Additional file 5: Table S2.** The gene information of different trends of Plant-pathogen interaction, Plant hormone signal transduction and MAPK signaling pathway.

**Additional file 6: Fig. S4.** The related genes expression thermogram of plant hormone signal transduction pathway.

**Additional file 7: Table S3.** The primer information of 20 genes for qRT-PCR.

## Data Availability

All transcriptomic sequencing data associated with this study have been submitted to the China National Center for Bioinformation (CNCB) and can be found using accession number PRJCA009200 (https://ngdc.cncb.ac.cn/bioproject/browse/PRJCA009200).
