# Supplementary material for: Transcriptome analysis reveals key genes involved in the resistance to Cryphonectria parasitica during early disease development in Chinese chestnut
Source: BMC Plant Biol. 2023 Feb 6;23:79. doi: 10.1186/s12870-023-04072-7 (PMC9901152; doi:10.1186/s12870-023-04072-7)
Supplement: Supplementary file 1 — Additional file 1: Fig. S1. Morphological analysis of inoculated (T) and non-inoculated (Mock) wounds of branches at 3 h, 6 h, 9 h and 12 h through stereomicroscopy. [file 12870_2023_4072_MOESM1_ESM.docx]

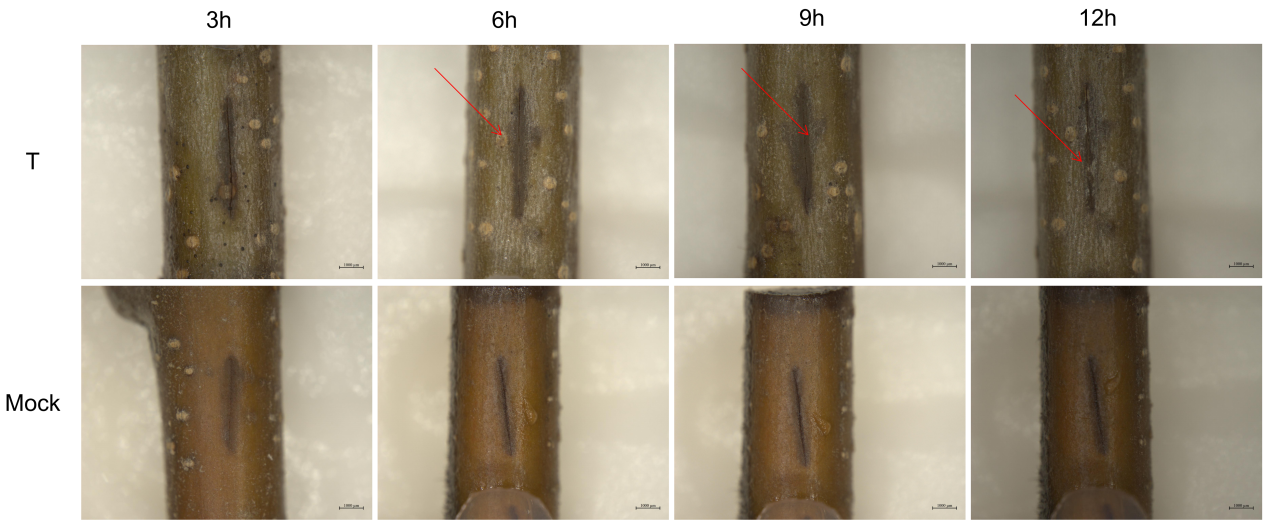


**Additional file 1: Figure. S1** Morphological analysis of inoculated (T) and non-inoculated (Mock) wounds of branches at 3h, 6h, 9h and 12h through stereomicroscopy.
