# Supplementary material for: Transcriptome analysis reveals key genes involved in the resistance to Cryphonectria parasitica during early disease development in Chinese chestnut
Source: BMC Plant Biol. 2023 Feb 6;23:79. doi: 10.1186/s12870-023-04072-7 (PMC9901152; doi:10.1186/s12870-023-04072-7)
Supplement: Supplementary file 2 — Additional file 2: Table. S1. Data quality assessment of each sample. [file 12870_2023_4072_MOESM2_ESM.docx]

**Additional file 2: Table. S1** Data quality assessment of each sample.

| Sample Name | Clean data | Mapping Reads | Q20 | Q30 | GC Content(%) | Reads aligned(%) |
| --- | --- | --- | --- | --- | --- | --- |
| Mock0h-1 | 54850433 | 50696564 | 97.74% | 93.41% | 43.87% | 92.43% |
| Mock0h-2 | 73838792 | 67791043 | 97.72% | 93.34% | 43.79% | 91.81% |
| Mock0h-3 | 68051165 | 62514322 | 97.62% | 93.12% | 43.85% | 91.86% |
| Mock3h-1 | 65505429 | 60979936 | 97.89% | 93.76% | 43.57% | 93.09% |
| Mock3h-2 | 65996961 | 60687669 | 97.73% | 93.39% | 43.60% | 91.96% |
| Mock3h-3 | 75179530 | 69134809 | 97.77% | 93.41% | 43.59% | 91.96% |
| Mock9h-1 | 53296068 | 49229354 | 97.69% | 93.31% | 43.78% | 92.37% |
| Mock9h-2 | 74893921 | 69147596 | 97.73% | 93.39% | 43.75% | 92.33% |
| Mock9h-3 | 78539927 | 72827639 | 97.83% | 93.60% | 43.97% | 92.73% |
| T3h-1 | 81324466 | 73109647 | 97.63% | 93.19% | 43.81% | 89.90% |
| T3h-2 | 66860495 | 61950408 | 97.70% | 93.35% | 43.94% | 92.66% |
| T3h-3 | 74804669 | 69216959 | 97.61% | 93.18% | 43.93% | 92.53% |
| T9h-1 | 72899194 | 63397476 | 97.65% | 93.18% | 44.68% | 86.97% |
| T9h-2 | 79309858 | 68874808 | 97.65% | 93.25% | 44.84% | 86.84% |
| T9h-3 | 92557015 | 81053938 | 97.83% | 93.56% | 44.38% | 87.57% |
