# Supplementary figures and images for: Transcriptome analysis reveals key genes involved in the resistance to Cryphonectria parasitica during early disease development in Chinese chestnut
Source: BMC Plant Biol. 2023 Feb 6;23:79. doi: 10.1186/s12870-023-04072-7 (PMC9901152; doi:10.1186/s12870-023-04072-7)

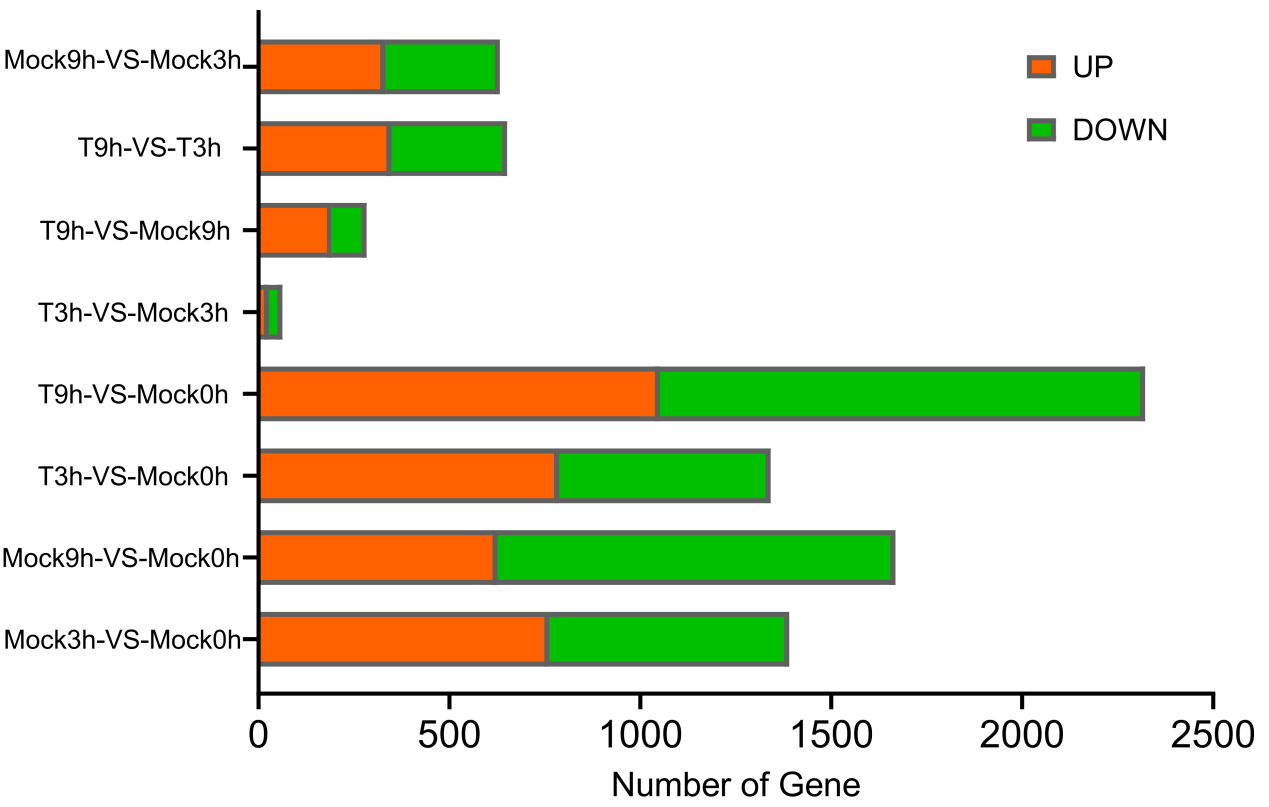


**Additional file 3: Figure. S2** Differentially expressed genes (DEGs) at different time points.

Supplement: Supplementary file 3 — Additional file 3: Fig. S2. Differentially expressed genes (DEGs) at different time points. [file 12870_2023_4072_MOESM3_ESM.docx]
