# Supplementary material for: Transcriptome analysis reveals key genes involved in the resistance to Cryphonectria parasitica during early disease development in Chinese chestnut
Source: BMC Plant Biol. 2023 Feb 6;23:79. doi: 10.1186/s12870-023-04072-7 (PMC9901152; doi:10.1186/s12870-023-04072-7)
Supplement: Supplementary file 4 — Additional file 4: Fig. S3. Differentially expressed genes (DEGs) at different trend characteristics at Mock0h-T3 h-T9 h. a: The genes showing an upward trend were annotated in KEGG; b: The genes showing an downward trend were annotated in KEGG. [file 12870_2023_4072_MOESM4_ESM.docx]

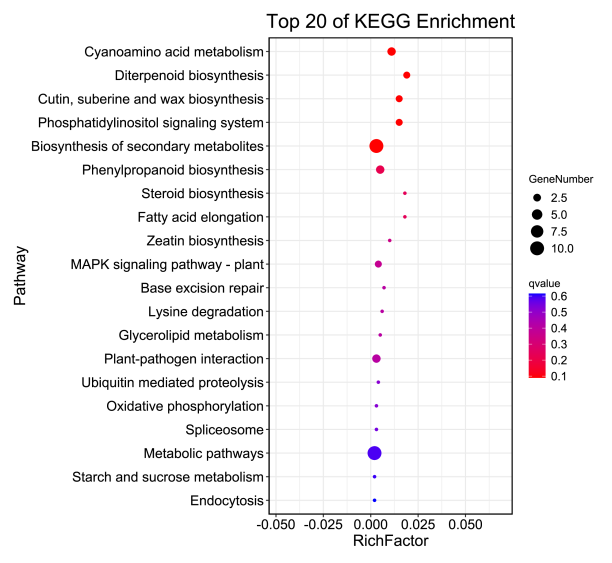

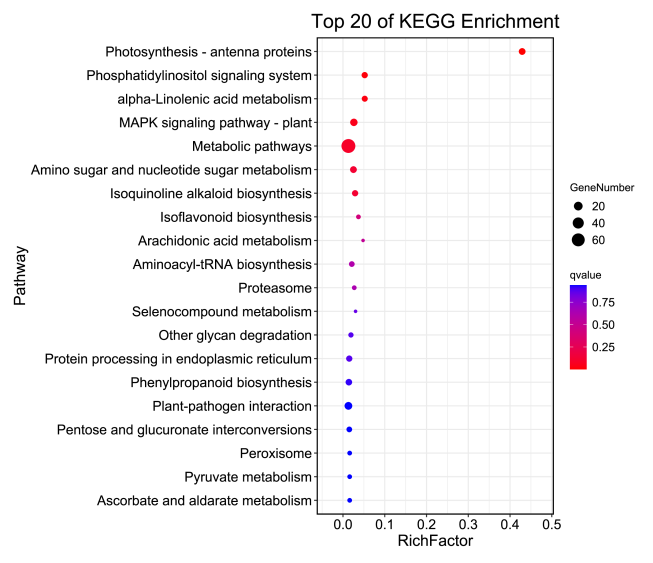


**Additional file 4: Figure. S3** Differentially expressed genes (DEGs) at different trend characteristics at Mock0h-T3h-T9h. a: The genes showing an upward trend were annotated in KEGG; b: The genes showing an downward trend were annotated in KEGG.
