# Supplementary material for: Transcriptome analysis reveals key genes involved in the resistance to Cryphonectria parasitica during early disease development in Chinese chestnut
Source: BMC Plant Biol. 2023 Feb 6;23:79. doi: 10.1186/s12870-023-04072-7 (PMC9901152; doi:10.1186/s12870-023-04072-7)
Supplement: Supplementary file 5 — Additional file 5: Table S2. The gene information of different trends of Plant-pathogen interaction, Plant hormone signal transduction and MAPK signaling pathway. [file 12870_2023_4072_MOESM5_ESM.docx]

Table S2 The gene information of different trends of Plant-pathogen interaction, Plant hormone signal transduction and MAPK signaling pathway

| Path way of upward trend | Gene number | Gene ID | gene annotation |
| --- | --- | --- | --- |
| Plant-pathogen interaction | 41 | CMHBY201980 | PREDICTED: probable cyclic nucleotide-gated ion channel 14-like [Glycine max] |
|  |  | CMHBY202144 | PREDICTED: probable WRKY transcription factor 31 [Nelumbo nucifera] |
|  |  | CMHBY202588 | PREDICTED: uncharacterized protein LOC100261821 [Vitis vinifera] |
|  |  | CMHBY202913 | PREDICTED: heat shock protein 83 [Jatropha curcas] |
|  |  | CMHBY205649 | Calcium dependent protein kinase 1 [Theobroma cacao] |
|  |  | CMHBY207153 | PREDICTED: probable calcium-binding protein CML48 [Jatropha curcas] |
|  |  | CMHBY207206 | PREDICTED: WRKY transcription factor 55-like [Populus euphratica] |
|  |  | CMHBY207599 | calmodulin [Quercus petraea] |
|  |  | CMHBY209375 | PREDICTED: probable WRKY transcription factor 75 [Populus euphratica] |
|  |  | CMHBY210024 | Calmodulin-like 11, putative [Theobroma cacao] |
|  |  | CMHBY212255 | PREDICTED: probable LRR receptor-like serine/threonine-protein kinase At1g51880 isoform X1 [Prunus mume] |
|  |  | CMHBY213153 | PREDICTED: putative receptor-like protein kinase At3g47110 [Fragaria vesca subsp. vesca] |
|  |  | CMHBY213392 | PREDICTED: elongation factor Tu, mitochondrial [Malus domestica] |
|  |  | CMHBY216663 | hypothetical protein EUTSA_v10012801mg [Eutrema salsugineum] |
|  |  | CMHBY216890 | Serine-threonine protein kinase, plant-type, putative [Theobroma cacao] |
|  |  | CMHBY216960 | NADPH:quinone oxidoreductase isoform 1 [Theobroma cacao] |
|  |  | CMHBY217023 | PREDICTED: probable LRR receptor-like serine/threonine-protein kinase At3g47570 isoform X1 [Malus domestica] |
|  |  | CMHBY217264 | WRKY DNA-binding protein 72, putative [Theobroma cacao] |
|  |  | CMHBY217617 | PREDICTED: probable disease resistance protein At4g27220 [Vitis vinifera] |
|  |  | CMHBY217635 | PREDICTED: putative leucine-rich repeat receptor-like protein kinase At2g19210-like isoform X1 [Citrus sinensis] |
|  |  | CMHBY217664 | PREDICTED: cyclic nucleotide-gated ion channel 1 [Tarenaya hassleriana] |
|  |  | CMHBY218069 | PREDICTED: cyclic nucleotide-gated ion channel 1-like [Pyrus x bretschneideri] |
|  |  | CMHBY219061 | Calcium-binding EF-hand family protein [Theobroma cacao] |
|  |  | CMHBY220547 | hypothetical protein PRUPE_ppa001427mg [Prunus persica] |
|  |  | CMHBY220713 | PREDICTED: basic form of pathogenesis-related protein 1-like [Citrus sinensis] |
|  |  | CMHBY220716 | Pathogenesis-related protein 1 [Theobroma cacao] |
|  |  | CMHBY220718 | pathogenesis-related protein 1 [Vitis hybrid cultivar] |
|  |  | CMHBY220835 | Calmodulin like 42 [Theobroma cacao] |
|  |  | CMHBY224029 | PREDICTED: probable calcium-binding protein CML18 [Solanum lycopersicum] |
|  |  | CMHBY225139 | hypothetical protein PRUPE_ppa023852mg, partial [Prunus persica] |
|  |  | CMHBY225579 | hypothetical protein CISIN_1g046398mg [Citrus sinensis] |
|  |  | CMHBY228970 | PREDICTED: probable calcium-binding protein CML30 [Vitis vinifera] |
|  |  | CMHBY229962 | PREDICTED: LRR receptor-like serine/threonine-protein kinase FLS2-like [Cicer arietinum] |
|  |  | CMHBY230167 | PREDICTED: LRR receptor-like serine/threonine-protein kinase GSO1 [Prunus mume] |
|  |  | CMHBY230298 | hypothetical protein B456_N013500 [Gossypium raimondii] |
|  |  | CMHBY231032 | PREDICTED: putative leucine-rich repeat receptor-like serine/threonine-protein kinase At2g19230 [Cucumis sativus] |
|  |  | CMHBY231395 | PREDICTED: ethylene-responsive transcription factor ERF096-like [Eucalyptus grandis] |
|  |  | CMHBY231483 | PREDICTED: probable disease resistance protein At1g12280 [Jatropha curcas] |
|  |  | CMHBY232735 | PREDICTED: probable LRR receptor-like serine/threonine-protein kinase At3g47570 [Fragaria vesca subsp. vesca] |
|  |  | CMHBY233937 | PREDICTED: probable leucine-rich repeat receptor-like serine/threonine-protein kinase At3g14840 [Prunus mume] |
|  |  | CMHBY235010 | PREDICTED: probable disease resistance protein At4g27220 [Vitis vinifera] |
|  |  | CMHBY235010 | PREDICTED: probable disease resistance protein At4g27220 [Vitis vinifera] |
| Plant hormone signal transduction | 18 | CMHBY203206 | PREDICTED: transcription factor SPATULA isoform X1 [Prunus mume] |
|  |  | CMHBY205871 | two-component response regulator [Rosa canina] |
|  |  | CMHBY216983 | BZIP transcription factor family protein [Theobroma cacao] |
|  |  | CMHBY220547 | hypothetical protein PRUPE_ppa001427mg [Prunus persica] |
|  |  | CMHBY220713 | PREDICTED: basic form of pathogenesis-related protein 1-like [Citrus sinensis] |
|  |  | CMHBY220716 | Pathogenesis-related protein 1 [Theobroma cacao] |
|  |  | CMHBY220718 | pathogenesis-related protein 1 [Vitis hybrid cultivar] |
|  |  | CMHBY221521 | putative esterase family protein [Populus trichocarpa] |
|  |  | CMHBY223154 | PREDICTED: indole-3-acetic acid-induced protein ARG7-like [Malus domestica] |
|  |  | CMHBY223599 | AP2/ERF domain-containing transcription factor, putative [Theobroma cacao] |
|  |  | CMHBY226291 | PREDICTED: probable carboxylesterase 6 [Vitis vinifera] |
|  |  | CMHBY226750 | Calmodulin binding protein, putative [Theobroma cacao] |
|  |  | CMHBY226753 | PREDICTED: uncharacterized protein LOC103964074 [Pyrus x bretschneideri] |
|  |  | CMHBY226755 | Calmodulin binding protein, putative [Theobroma cacao] |
|  |  | CMHBY228632 | PREDICTED: abscisic acid receptor PYL4 [Nelumbo nucifera] |
|  |  | CMHBY231386 | PREDICTED: abscisic acid receptor PYL2 [Jatropha curcas] |
|  |  | CMHBY232135 | PREDICTED: probable carboxylesterase 18 [Jatropha curcas] |
|  |  | CMHBY232165 | hypothetical protein PRUPE_ppa026008mg [Prunus persica] |
| MAPK signaling pathway - plant | 20 | CMHBY202144 | PREDICTED: probable WRKY transcription factor 31 [Nelumbo nucifera] |
|  |  | CMHBY207599 | calmodulin [Quercus petraea] |
|  |  | CMHBY209375 | PREDICTED: probable WRKY transcription factor 75 [Populus euphratica] |
|  |  | CMHBY212255 | PREDICTED: probable LRR receptor-like serine/threonine-protein kinase At1g51880 isoform X1 [Prunus mume] |
|  |  | CMHBY217264 | WRKY DNA-binding protein 72, putative [Theobroma cacao] |
|  |  | CMHBY217635 | PREDICTED: putative leucine-rich repeat receptor-like protein kinase At2g19210-like isoform X1 [Citrus sinensis] |
|  |  | CMHBY220547 | hypothetical protein PRUPE_ppa001427mg [Prunus persica] |
|  |  | CMHBY220713 | PREDICTED: basic form of pathogenesis-related protein 1-like [Citrus sinensis] |
|  |  | CMHBY220716 | Pathogenesis-related protein 1 [Theobroma cacao] |
|  |  | CMHBY220718 | pathogenesis-related protein 1 [Vitis hybrid cultivar] |
|  |  | CMHBY223599 | AP2/ERF domain-containing transcription factor, putative [Theobroma cacao] |
|  |  | CMHBY225139 | hypothetical protein PRUPE_ppa023852mg, partial [Prunus persica] |
|  |  | CMHBY225579 | hypothetical protein CISIN_1g046398mg [Citrus sinensis] |
|  |  | CMHBY228632 | PREDICTED: abscisic acid receptor PYL4 [Nelumbo nucifera] |
|  |  | CMHBY228970 | PREDICTED: probable calcium-binding protein CML30 [Vitis vinifera] |
|  |  | CMHBY229962 | PREDICTED: LRR receptor-like serine/threonine-protein kinase FLS2-like [Cicer arietinum] |
|  |  | CMHBY230167 | PREDICTED: LRR receptor-like serine/threonine-protein kinase GSO1 [Prunus mume] |
|  |  | CMHBY231032 | PREDICTED: putative leucine-rich repeat receptor-like serine/threonine-protein kinase At2g19230 [Cucumis sativus] |
|  |  | CMHBY231386 | PREDICTED: abscisic acid receptor PYL2 [Jatropha curcas] |
|  |  | CMHBY233937 | PREDICTED: probable leucine-rich repeat receptor-like serine/threonine-protein kinase At3g14840 [Prunus mume] |
| Path way of downward tren | Gene number | Gene ID | gene annotation |
| MAPK signaling pathway - plant | 12 | CMHBY204845 | respiratory burst oxidase, putative [Ricinus communis] |
|  |  | CMHBY209375 | PREDICTED: probable WRKY transcription factor 75 [Populus euphratica] |
|  |  | CMHBY217264 | WRKY DNA-binding protein 72, putative [Theobroma cacao] |
|  |  | CMHBY220716 | Pathogenesis-related protein 1 [Theobroma cacao] |
|  |  | CMHBY224655 | hypothetical protein JCGZ_04288 [Jatropha curcas] |
|  |  | CMHBY224656 | hypothetical protein VITISV_023174 [Vitis vinifera] |
|  |  | CMHBY224658 | hypothetical protein VITISV_023174 [Vitis vinifera] |
|  |  | CMHBY224659 | hypothetical protein JCGZ_04288 [Jatropha curcas] |
|  |  | CMHBY224660 | hypothetical protein VITISV_023174 [Vitis vinifera] |
|  |  | CMHBY224661 | Calcium-binding EF-hand family protein, putative [Theobroma cacao] |
|  |  | CMHBY224662 | Calcium-binding EF-hand family protein, putative [Theobroma cacao] |
|  |  | CMHBY231394 | PREDICTED: ethylene-responsive transcription factor ERF098 [Fragaria vesca subsp. vesca] |
| Plant-pathogen interaction | 14 | CMHBY204845 | respiratory burst oxidase, putative [Ricinus communis] |
|  |  | CMHBY209375 | PREDICTED: probable WRKY transcription factor 75 [Populus euphratica] |
|  |  | CMHBY209582 | hypothetical protein POPTR_0012s01380g [Populus trichocarpa] |
|  |  | CMHBY217264 | WRKY DNA-binding protein 72, putative [Theobroma cacao] |
|  |  | CMHBY220716 | Pathogenesis-related protein 1 [Theobroma cacao] |
|  |  | CMHBY220835 | Calmodulin like 42 [Theobroma cacao] |
|  |  | CMHBY224655 | hypothetical protein JCGZ_04288 [Jatropha curcas] |
|  |  | CMHBY224656 | hypothetical protein VITISV_023174 [Vitis vinifera] |
|  |  | CMHBY224658 | hypothetical protein VITISV_023174 [Vitis vinifera] |
|  |  | CMHBY224659 | hypothetical protein JCGZ_04288 [Jatropha curcas] |
|  |  | CMHBY224660 | hypothetical protein VITISV_023174 [Vitis vinifera] |
|  |  | CMHBY224661 | Calcium-binding EF-hand family protein, putative [Theobroma cacao] |
|  |  | CMHBY224662 | Calcium-binding EF-hand family protein, putative [Theobroma cacao] |
|  |  | CMHBY229455 | hypothetical protein VITISV_007397 [Vitis vinifera] |
| Plant hormone signal transduction | 7 | CMHBY201184 | Gibberellin receptor GID1, putative [Ricinus communis] |
|  |  | CMHBY220716 | Pathogenesis-related protein 1 [Theobroma cacao] |
|  |  | CMHBY221333 | Carboxylesterase 1 [Morus notabilis] |
|  |  | CMHBY223154 | PREDICTED: indole-3-acetic acid-induced protein ARG7-like [Malus domestica] |
|  |  | CMHBY226751 | Calmodulin binding protein, putative [Theobroma cacao] |
|  |  | CMHBY226755 | Calmodulin binding protein, putative [Theobroma cacao] |
|  |  | CMHBY231394 | PREDICTED: ethylene-responsive transcription factor ERF098 [Fragaria vesca subsp. vesca] |
