# Supplementary material for: Transcriptome analysis reveals key genes involved in the resistance to Cryphonectria parasitica during early disease development in Chinese chestnut
Source: BMC Plant Biol. 2023 Feb 6;23:79. doi: 10.1186/s12870-023-04072-7 (PMC9901152; doi:10.1186/s12870-023-04072-7)
Supplement: Supplementary file 6 — Additional file 6: Fig. S4. The related genes expression thermogram of plant hormone signal transduction pathway. [file 12870_2023_4072_MOESM6_ESM.docx]

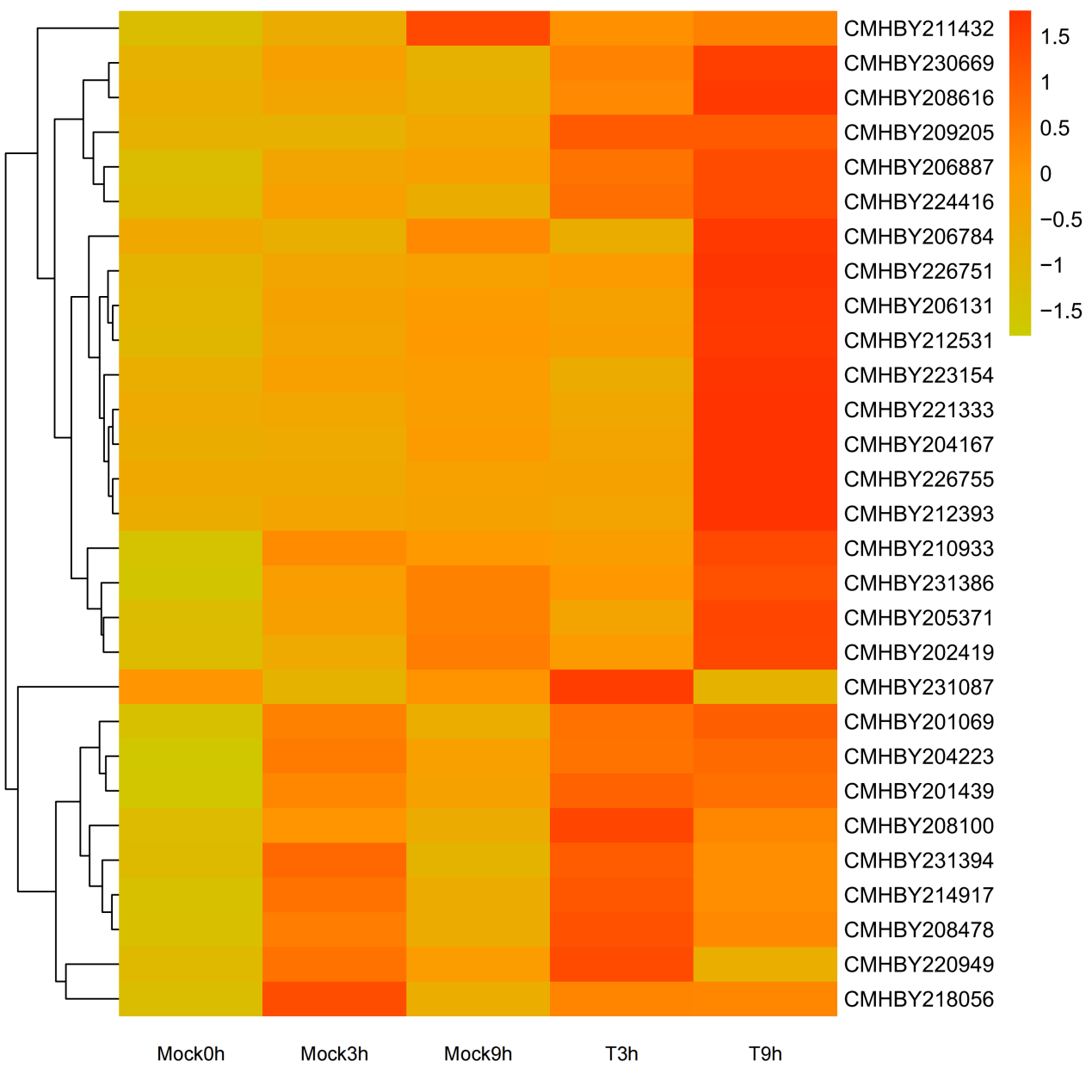


**Additional file 6: Figure. S4** The related genes expression thermogram of [plant hormone signal transduction](file:///C:\Users\ASUS\AppData\Local\Temp\360zip$Temp\360$1\koenrich674c33\out\5021644996543.os\KO\out.htm#gene28) pathway.
