# Supplementary material for: Transcriptome analysis reveals key genes involved in the resistance to Cryphonectria parasitica during early disease development in Chinese chestnut
Source: BMC Plant Biol. 2023 Feb 6;23:79. doi: 10.1186/s12870-023-04072-7 (PMC9901152; doi:10.1186/s12870-023-04072-7)
Supplement: Supplementary file 7 — Additional file 7: Table S3. The primer information of 20 genes for qRT-PCR. [file 12870_2023_4072_MOESM7_ESM.docx]

**Additional file 7: Table. S3** The primer information of 20 genes for qRT-PCR.

| Gene ID | Left Primer | Right Primer |
| --- | --- | --- |
| CMHBY217011 | CAACCGAAGCGTGGTAGATG | AGGTGTCTTGGTACGCTTCA |
| CMHBY231394 | AGGCAAAGGAAGAGGTGAGA | TGCCTTGAAGGGTCTCGAAT |
| CMHBY203204 | CTCCGGGAATTGACAGCAAG | CCAGTGTAGACGCCGATTTC |
| CMHBY224416 | CTTCTTCAGCTGATGGCAAGA | TTGCCAAACCCTTTGCCTAC |
| CMHBY202559 | GGGTTCCATGTATGCTGCTG | ACATCCCAGCTGCTCTTTCT |
| CMHBY202677 | ATGGCTGCCTTCTTAGGTGT | GCAGGCCTTGCCACATATAG |
| CMHBY230316 | GGTGATTTGGACGGTGATGG | CCTCCTCAAGCCAGAACTGA |
| CMHBY203546 | TAAAGGAAGAGGCCGAAGCA | CACAGCGAGCAAGAGAGTTC |
| CMHBY210481 | CGGAGACTACTTTCCCGACA | TGGCCACCCAGTTACTACAG |
| CMHBY212255 | AGTTGGGAAGCGAGACTTCA | AGTGCCATCATCAGTTGGGA |
| CMHBY215114 | TGCTAGACACAATGGACCGT | GTCCAGGCATGTCAAACCTC |
| CMHBY225337 | TGATGCTCCCATCTGAGGAC | CAGGCACGTCCAAGGTTATG |
| CMHBY230281 | TCAAGATGGATGTGCCTGGT | CTCTCTCCGCTCACTCTCAG |
| CMHBY204223 | AACAAACGGCAACGGTACAA | TCCTTCTGCTAGCCCTGATG |
| CMHBY206887 | TGTTGTGAAGACCGGACTGA | TATCAGCCGCAACGATCAGA |
| CMHBY211432 | GCTTCAAGTTCAACGACGGA | GCGAGTAGGGTTGAGCATTG |
| CMHBY231386 | ACCTTCAGAAGCTTGCCTCA | CCACCACTGCAACCATCAAA |
| CMHBY228079 | AAGCTTCTCTGCAGGCATTG | TTCCAGTTTGGCGTTCCAAG |
| CMHBY219172 | TACGGTGTTACCACTGGCTT | GTTTGCGCGAACAAGCATAG |
| CMHBY227993 | GAGCTCACCCAGGTTCGATA | CAATGCTTCCACCTCCTTCG |
